# Effects of physical exercise on the happiness of Chinese university students: the moderated mediation model of grit through peer relationship and hope

**DOI:** 10.3389/fpsyg.2026.1822698

**Published:** 2026-05-22

**Authors:** Jin Chen

**Affiliations:** Nanjing Vocational College of Information Technology, Nanjing, China

**Keywords:** conditional indirect effect, grit, happiness, peer relationship, physical exercise

## Abstract

This study examined whether grit moderates the serial mediating effects of peer relationships and hope in the association between physical exercise and happiness among Chinese university students. The primary aim was to generate preliminary empirical evidence that may inform the development of intervention strategies designed to enhance hope and psychological wellbeing in emerging adulthood. Participants were recruited using a multistage stratified sampling strategy, followed by a census-based data collection approach in which all eligible students within the selected departments were surveyed. This process resulted in a final sample of 508 undergraduate students. Data were analyzed using IBM SPSS Statistics 25.0, AMOS 23.0, and the PROCESS macro (Version 4.2). The analytical procedures included descriptive statistics, reliability testing, exploratory and confirmatory factor analyses, measurement model evaluation, and moderated mediation analysis using PROCESS Model 83 with bootstrapping. The results yielded two principal findings. First, physical exercise, grit, peer relationships, hope, and happiness were positively and significantly intercorrelated. Second, grit demonstrated a significant conditional indirect effect in the pathway from physical exercise to happiness via peer relationships and hope. Specifically, the conditional indirect effect was stronger at higher levels of grit, indicating that grit amplifies the positive association between physical exercise and happiness. These findings contribute preliminary evidence to the growing literature on grit by identifying its enhancing role within a positive psychological process model. Rather than functioning solely as a protective buffer, grit appears to strengthen the beneficial pathway linking health-promoting behaviors to interpersonal and cognitive-emotional resources, ultimately fostering happiness among Chinese university students.

## 1 Introduction

University students experience a critical developmental transition characterized by increased academic demands, social reorganization, and identity formation. During this period, psychological wellbeing becomes both a developmental outcome and a protective factor against maladjustment. Happiness, conceptualized as a cognitive–affective evaluation of life, has been widely recognized as a key indicator of successful adaptation in emerging adulthood (Diener et al., 2018). Identifying factors that enhance happiness among university students is therefore of substantial theoretical and practical importance.

The present study is primarily grounded in social integration theory, which emphasizes the fundamental role of interpersonal relationships in promoting psychological wellbeing (Baumeister and Leary, 1995). From this perspective, individuals derive emotional stability and life satisfaction through meaningful social connections. In the context of university life, peer relationships represent a central source of social integration and may serve as a key mechanism linking behavioral engagement to psychological outcomes.

Physical exercise has consistently been associated with improved psychological wellbeing. Beyond its physiological benefits, physical exercise contributes to emotional regulation, stress reduction, and social integration (Biddle and Asare, 2011; Eime et al., 2013). Importantly, in university settings, physical exercise often occurs in peer-based contexts, suggesting that the association with wellbeing may operate through enhanced peer relationships. Consistent with social integration theory, supportive peer relationships have been shown to promote life satisfaction and resilience (Rueger et al., 2016). However, prior research has largely examined peer relationships as a direct predictor or simple mediator, without fully considering the underlying cognitive-motivational processes.

Within this framework, hope represents a key cognitive-motivational mechanism through which social and behavioral experiences are translated into psychological wellbeing. Hope, defined as a goal-directed cognitive set comprising agency and pathways (Snyder, 2002), has been consistently linked to positive affect and life satisfaction. Individuals embedded in supportive peer environments are more likely to develop optimistic expectations and sustained goal pursuit, thereby enhancing hope (Gallagher and Lopez, 2009; Marques et al., 2011). In turn, elevated levels of hope contribute to greater happiness. Despite this, limited research has examined hope as a sequential mediator linking physical exercise and peer relationships to happiness.

In addition to these mechanisms, individual differences may shape the strength of these relationships. Grit, defined as perseverance and passion for long-term goals (Duckworth et al., 2007), has been associated with resilience and wellbeing, although its effects vary across contexts (Credé et al., 2017). Emerging evidence suggests that grit may function as a moderator within social-cognitive frameworks, strengthening the extent to which individuals benefit from behavioral and social experiences (Lan and Radin, 2020). However, its role within a moderated serial mediation model linking physical exercise, peer relationships, hope, and happiness remains largely underexplored, particularly within an integrated framework.

These relationships may be particularly salient in the Chinese cultural context, which is characterized by collectivistic values emphasizing interpersonal harmony, social connectedness, and group cohesion. In such contexts, peer relationships play a more central role in shaping psychological experiences compared to individualistic societies. Strong and supportive peer networks not only facilitate social integration but also provide essential emotional and motivational resources.

Moreover, in collectivistic cultures, hope is less likely to be constructed solely as an individual cognitive resource and more likely to be related to relational contexts and social support systems. That is, individuals may derive their sense of agency and goal-directed pathways not only from personal competence but also from encouragement, expectations, and validation within their social networks.

Similarly, happiness in collectivistic settings is often closely tied to relational harmony and social functioning rather than purely individual achievement or personal satisfaction. Therefore, the associations among physical exercise, peer relationship, hope, and happiness may be particularly pronounced and interdependent in the Chinese context. This cultural perspective underscores the importance of examining these variables within an integrated and contextually grounded framework, particularly in understanding how interpersonal, cognitive, and behavioral processes jointly operate in collectivistic settings.

Despite these theoretical and empirical advances, no study has simultaneously examined the moderated serial mediation process linking physical exercise, peer relationship, hope, and happiness within a single integrated framework. This gap limits a comprehensive understanding of how behavioral engagement, social relationships, cognitive-motivational processes, and personality traits jointly contribute to psychological wellbeing.

While prior studies have identified peer relationships and hope as important predictors of psychological wellbeing, the present study argues that these constructs are not merely parallel influences but are theoretically linked in a sequential process. Grounded in social integration theory, peer relationships provide foundational social and emotional resources that foster a sense of belonging and stability. Building on hope theory, hope is conceptualized as a goal-directed cognitive-motivational system that develops based on such underlying psychosocial conditions. Therefore, hope is more appropriately understood as emerging from prior social experiences rather than functioning independently, supporting a serial mediation framework.

Furthermore, grit is conceptualized not as an intervening mechanism but as a boundary condition within this process. Given its trait-like nature, grit is relatively stable and unlikely to be directly shaped by peer relationships or physical exercise. Instead, grit influences the extent to which individuals translate social and cognitive resources into psychological wellbeing. Accordingly, grit is positioned as a moderator that conditions the strength of the indirect effects, rather than as an alternative mediator. Importantly, university students represent an appropriate population for examining these relationships due to their developmental characteristics. Emerging adulthood involves significant transitions in identity formation, autonomy, and social relationships (Arnett, 2000). During this period, physical exercise is often embedded in peer contexts, while cognitive-motivational resources such as hope and personality traits such as grit are actively developed, influencing how individuals translate experiences into psychological wellbeing.

Accordingly, the present study proposes a moderated serial mediation model in which peer relationship and hope sequentially mediate the association between physical exercise and happiness, and grit moderates this process among Chinese university students. By anchoring the model in social integration theory and integrating cognitive-motivational and personality factors, this study provides a more comprehensive understanding of the mechanisms through which physical exercise contributes to psychological wellbeing.

## 2 Theoretical background

### 2.1 Relationship between physical exercise and happiness

Physical exercise is commonly conceptualized as planned, structured, and repetitive bodily movement performed with the objective of improving or maintaining physical fitness (Caspersen et al., 1985). More broadly, it encompasses any bodily movement produced by skeletal muscles that is associated with energy expenditure (World Health Organization, 2020). This distinction is theoretically important, as physical exercise typically involves intentional engagement, goal orientation, and sustained participation, which may differentially be related to psychological outcomes among university students.

Happiness is most frequently examined within the broader framework of subjective wellbeing (SWB), which includes both cognitive evaluations of life satisfaction and affective components such as positive and negative effect (Diener, 1984). In this context, happiness reflects a relatively stable and multidimensional evaluation of one’s life, incorporating emotional experiences, life satisfaction, and a sense of meaning (Diener et al., 2018). Among university students, happiness serves as a key indicator of psychological adjustment during a period characterized by academic demands, identity formation, and evolving social relationships.

The relationship between physical exercise and happiness can be explained through multiple mechanisms. Physiologically, exercise contributes to mood regulation and stress reduction. Psychologically, it enhances self-efficacy, perceived competence, and positive self-evaluations. Socially, physical exercise—particularly in university contexts—often occurs in peer-based settings such as sports teams and group activities, fostering social connectedness and belongingness (Eime et al., 2013). From a social integration perspective, these interpersonal experiences may serve as a key pathway through which physical exercise contributes to psychological wellbeing.

A substantial body of empirical research supports a positive association between physical exercise and happiness. A systematic review by Zhang and Chen (2019) reported that most studies identify a positive relationship between exercise and happiness, highlighting mediating roles of perceived health and social engagement. Similarly, a meta-analysis by Buecker et al. (2021) found a significant overall association between physical exercise and subjective wellbeing, suggesting that this relationship is robust across diverse samples and methodological approaches.

Evidence from university student populations further supports this relationship. For instance, Zhang et al. (2021) found that physical exercise was positively associated with subjective wellbeing among Chinese college students, with perceived health acting as a mediator. Jiang et al. (2021) reported similar findings, while also noting gender differences in the strength of the association. At a broader level, Pengpid and Peltzer (2019) demonstrated that physical exercise was positively related to happiness and life satisfaction among university students across 24 countries, indicating cross-cultural generalizability.

However, the relationship between physical exercise and happiness is not uniformly consistent. Some studies have reported variability in effect sizes depending on contextual and individual factors, such as social environment, motivation, and personal characteristics (Credé et al., 2017). These inconsistencies suggest that the effects of physical exercise may depend on underlying mechanisms and boundary conditions rather than operating as a simple direct relationship.

Collectively, while existing research provides strong support for a positive association between physical exercise and happiness, much of the literature relies on cross-sectional designs or simple mediation models. As a result, less attention has been given to the complex interplay of relational and cognitive-motivational processes, as well as individual differences that may shape this relationship. Addressing these limitations may contribute to a more comprehensive understanding of how physical exercise promotes happiness among university students.

### 2.2 Serial mediating effects of peer relationship and hope between physical exercise and happiness

Understanding how physical exercise contributes to happiness requires identifying the interpersonal and cognitive mechanisms through which behavioral engagement translates into psychological wellbeing. Drawing on social integration theory and hope theory, the present framework proposes that peer relationship and hope sequentially mediate the association between physical exercise and happiness.

#### 2.2.1 Mediating effect of peer relationship

Peer relationship refers to the quality of interpersonal connections and mutual interactions with age-matched individuals, characterized by support, intimacy, trust, and reciprocity (Bukowski et al., 1994). It has also been conceptualized as a form of social support embedded within horizontal social networks, contributing to belongingness and social adjustment during adolescence and emerging adulthood (Hartup, 1996). In the university context, peer relationships serve as a primary source of emotional support, identity validation, and daily interaction.

From a social integration perspective, physical exercise may enhance peer relationships by creating structured opportunities for social interaction and shared experiences. In particular, participation in group-based or organized physical activities facilitates cooperation, communication, and collective goal pursuit, thereby strengthening interpersonal bonds. Activities such as sports teams, fitness classes, and recreational programs provide contexts for emotional exchange and social identity formation (Eime et al., 2013). Empirical evidence supports this view, indicating that adolescents and university students who engage more frequently in physical exercise report higher levels of peer connectedness and perceived social support (McMahon et al., 2017). Similarly, Richards et al. (2015) found that physically active individuals were more likely to experience positive social interactions and greater social integration. These findings suggest that physical exercise enhances peer relationships by fostering socially embedded experiences.

In turn, high-quality peer relationships contribute significantly to psychological wellbeing. The need-to-belong theory posits that stable and supportive interpersonal relationships are fundamental to human wellbeing (Baumeister and Leary, 1995). Consistent with this perspective, empirical research demonstrates that perceived peer support is positively associated with life satisfaction and subjective happiness among university students (Rueger et al., 2016). Moreover, Demir and Özdemir (2010) found that friendship quality predicts happiness above and beyond personality traits and demographic factors. These findings indicate that peer relationship serves as a key interpersonal mechanism linking physical exercise to happiness.

However, not all peer interactions are inherently beneficial. Some studies have shown that peer relationships may involve conflict, exclusion, or social pressure, which can undermine psychological wellbeing (Rueger et al., 2016). This suggests that the effects of peer relationships are contingent upon their quality and context, rather than being uniformly positive.

Taken together, while physical exercise is likely to promote peer relationships, and peer relationships are generally associated with greater happiness, the strength of these associations may vary depending on contextual and interpersonal factors. This highlights the importance of examining peer relationship as a mediating mechanism within a broader, conditionally specified model.

#### 2.2.2 Mediating effect of hope

Hope is defined as a positive motivational state based on two interacting components: agency (goal-directed determination) and pathways (planning to achieve goals) (Snyder, 2002). From this perspective, hope reflects an individual’s perceived capacity to initiate and sustain movement toward valued goals. Alternatively, hope has been conceptualized as a cognitive-emotional resource that enables adaptive coping and resilience in challenging circumstances (Gallagher and Lopez, 2009).

Within the framework of social integration, hope can be understood as a cognitive-motivational resource that develops from individuals’ behavioral and interpersonal experiences. Engagement in physical exercise may foster hope by enhancing self-regulatory capacity, mastery experiences, and perceived competence. Regular exercise provides repeated opportunities for goal setting, effort investment, and observable progress, thereby strengthening agency beliefs. Empirical studies indicate that higher levels of physical exercise are associated with greater psychological resilience and optimism, both closely related to hope (Herbert et al., 2020). In addition, Shang et al. (2021) demonstrated that physical exercise indirectly enhanced subjective wellbeing through improvements in self-esteem and body image, suggesting that exercise may cultivate positive self-perceptions that underpin hopeful thinking. These findings support the proposition that physical exercise contributes to higher levels of hope.

Hope has been consistently identified as a strong predictor of happiness and subjective wellbeing. According to hope theory, individuals high in hope are more likely to perceive life as meaningful and manageable, leading to greater life satisfaction and wellbeing (Snyder, 2002). Empirical evidence confirms that hope significantly predicts happiness across diverse populations (Gallagher and Lopez, 2009). Moreover, Marques et al. (2011) found that interventions designed to increase hope resulted in improvements in wellbeing and positive emotions. Thus, hope functions as a cognitive-motivational mechanism translating behavioral and relational experiences into sustained psychological wellbeing.

However, the effects of hope are not uniformly consistent across contexts. Some studies suggest that hope may be less predictive of wellbeing when individuals lack sufficient external resources or social support (Gallagher and Lopez, 2009). This indicates that hope does not operate in isolation but rather depends on the availability of supportive environmental and interpersonal conditions.

Taken together, while physical exercise is likely to enhance hope and hope is positively associated with happiness, its effects may be contingent upon relational contexts. This further supports the need to examine hope as part of a sequential and contextually embedded process, rather than as an independent mediator.

#### 2.2.3 Effect of peer relationship on hope

Beyond their independent mediating roles, peer relationship may is related to hope by shaping individuals’ motivational and cognitive resources. Social support theory suggests that supportive peer environments enhance individuals’ perceived coping capacity and goal attainability (Cohen and Wills, 1985). When students experience trust, encouragement, and affirmation within peer networks, they are more likely to develop stronger agency beliefs and confidence in achieving future goals.

Empirical research supports this linkage. Antonucci (2018) found that perceived peer support positively predicted levels of hope among adolescents. Similarly, Marques et al. (2011) demonstrated that relational encouragement and social reinforcement are critical components in fostering hopeful thinking. These findings indicate that peer relationship not only directly enhances happiness but also strengthens hope.

Importantly, the sequential ordering of peer relationship and hope is theoretically grounded. In university contexts, physical exercise is typically embedded in socially interactive settings (e.g., group activities, sports teams), making peer relationship a more immediate interpersonal outcome of behavioral engagement. In contrast, hope represents a subsequent cognitive-motivational state that develops from these social experiences. Supportive peer relationships provide emotional affirmation, encouragement, and goal-related feedback, which in turn strengthen individuals’ agency and pathways thinking. Therefore, peer relationship is conceptualized as an antecedent interpersonal resource, whereas hope functions as a downstream cognitive-motivational mechanism.

Taken together, theoretical reasoning and empirical evidence support a serial mediation model in which physical exercise enhances peer relationships, which in turn foster hope, ultimately contributing to greater happiness.

### 2.3 Moderating effect of grit on the relationship between physical exercise and peer relationship

Grit is conceptualized as a dispositional tendency reflecting sustained passion and perseverance toward long-term goals (Duckworth et al., 2007). It encompasses perseverance of effort and consistency of interests over time, emphasizing endurance rather than short-term intensity. Unlike transient motivation, grit captures an individual’s capacity to remain committed despite setbacks or challenges. Subsequent research has confirmed its multidimensional structure and its role as a non-cognitive resource contributing to adaptive functioning across domains (Duckworth and Quinn, 2009; Datu et al., 2021).

Although grit has often been conceptualized as a relatively stable personality trait, emerging evidence suggests that it is shaped by contextual experiences, including structured and goal-directed activities such as physical exercise. Regular participation in physical exercise requires persistence, delayed gratification, and sustained effort—behaviors closely aligned with the core components of grit. Empirical studies have shown that higher levels of physical exercise are associated with greater grit and resilience among university students (Dunston et al., 2022), and longitudinal evidence further suggests that exercise participation may contribute to the development of perseverance-related dispositions (Bae et al., 2024).

Beyond its intrapersonal role, grit has also been linked to interpersonal functioning. Individuals high in grit are more likely to maintain consistent engagement in social contexts, tolerate interpersonal difficulties, and persist in relationship-building efforts. Prior research indicates that grit is positively associated with peer relationship quality and social adjustment (Lan, 2020), and longitudinal studies suggest reciprocal relationships between grit and peer relationships over time (Cui and Yang, 2022). These findings imply that gritty individuals are more likely to invest sustained effort in maintaining and strengthening social ties.

From this perspective, grit is particularly relevant as a moderating factor in the relationship between physical exercise and peer relationships. Physical exercise provides opportunities for peer interaction through shared activities, cooperation, and collective goal pursuit. However, the extent to which these opportunities translate into meaningful and enduring peer relationships may depend on individuals’ perseverance and sustained engagement.

For individuals high in grit, exercise participation is more likely to involve consistent attendance, persistence in the face of challenges, and continued involvement in group activities. This sustained engagement increases repeated peer interaction, fostering trust, mutual understanding, and relational stability. In contrast, individuals low in grit may be more likely to disengage when encountering difficulties or social discomfort, thereby limiting the interpersonal benefits of exercise.

Thus, grit can be conceptualized as an amplifying condition under which the positive association between physical exercise and peer relationships becomes stronger. Specifically, the relationship between physical exercise and peer relationship quality is expected to be stronger among individuals with higher levels of grit, and weaker among those with lower levels of grit.

However, the role of grit is not uniformly robust. Meta-analytic evidence suggests that its predictive effects are often modest and context-dependent (Credé et al., 2017). This inconsistency indicates that grit may function more appropriately as a moderator rather than a direct predictor, shaping the conditions under which behavioral engagement translates into relational outcomes.

Taken together, grit is posited to function as a boundary condition that is related to how physical exercise contributes to peer relationship formation, thereby strengthening the conditional process underlying the proposed model.

### 2.4 The present study

The present study examines a moderated serial mediation model linking physical exercise to happiness among Chinese university students. Specifically, it investigates whether peer relationships and hope sequentially mediate the association between physical exercise and happiness, and whether grit moderates this process.

Drawing on prior theoretical and empirical research, physical exercise is expected to enhance psychological wellbeing through both interpersonal and cognitive-motivational mechanisms. Participation in physical exercise may promote peer relationships by providing opportunities for social interaction and shared experiences. In turn, positive peer relationships are likely to foster hope as a goal-directed cognitive resource, which has been consistently identified as an important predictor of happiness. Accordingly, physical exercise is expected to be positively associated with peer relationships, peer relationships with hope, and hope with happiness.

Furthermore, it is proposed that peer relationships and hope sequentially mediate the relationship between physical exercise and happiness, reflecting a chain of interpersonal and cognitive-motivational processes. In addition, individual differences in personality traits may influence the extent to which individuals benefit from these experiences. In particular, grit is expected to moderate the relationship between physical exercise and peer relationships, thereby influencing the strength of the indirect effect of physical exercise on happiness through peer relationships and hope.

**Figure 1:**
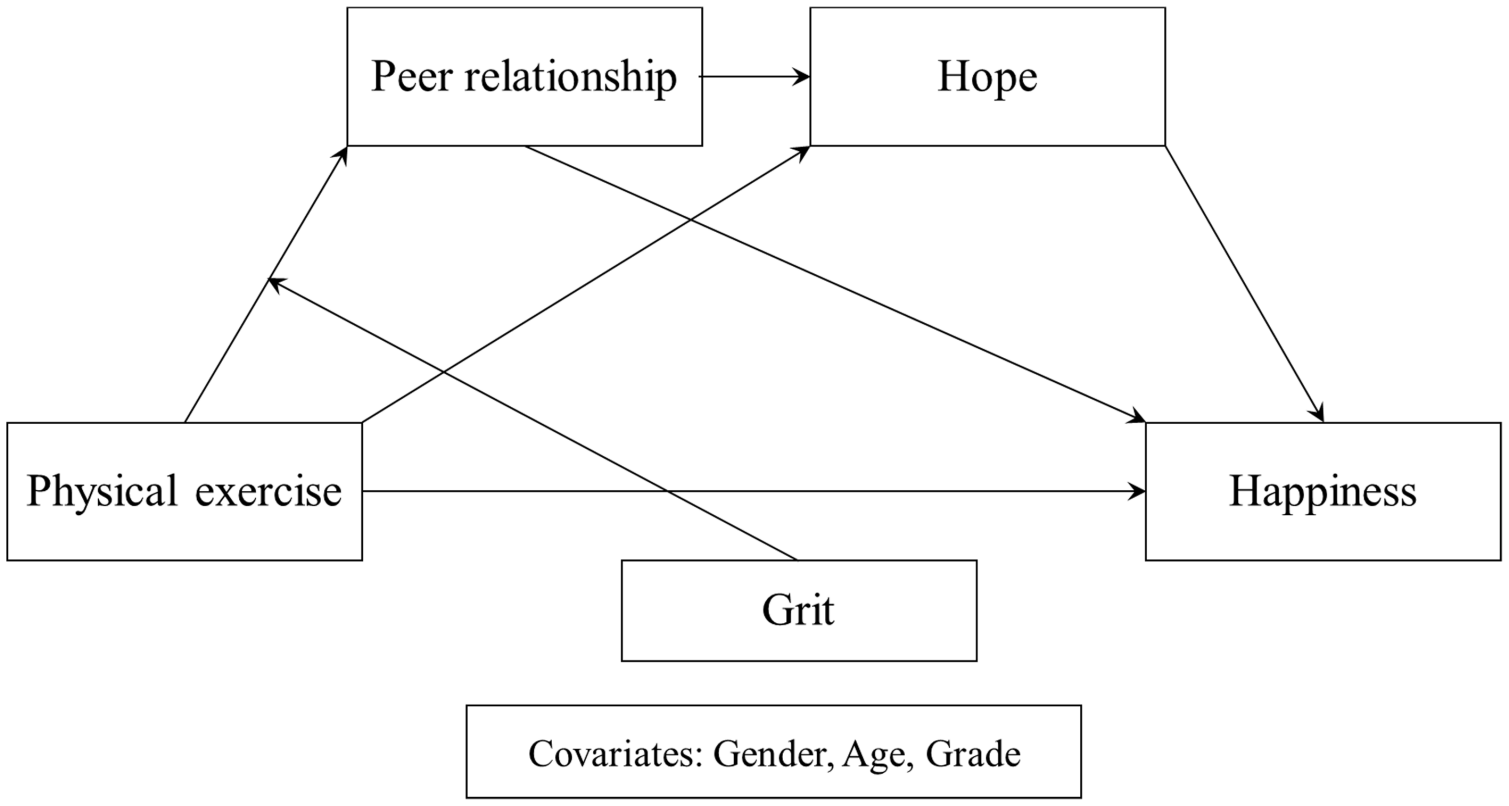
Conceptual research model.

Based on these assumptions, this study proposes a moderated serial mediation model in which physical exercise is specified as the independent variable and happiness as the dependent variable, with peer relationships and hope serving as sequential mediators, and grit moderating the relationship between physical exercise and peer relationships. The proposed model is presented in Figure 1.

## 3 Research methods

### 3.1 Participants and data collection method

This study employed a cross-sectional survey design guided by the Strengthening the Reporting of Observational Studies in Epidemiology (STROBE) guidelines. A multistage stratified sampling strategy was adopted to enhance geographic representativeness and minimize regional sampling bias across China.

To ensure balanced regional representation, five provinces were selected to reflect the eastern, western, southern, northern, and central regions of China. In the first stage, one province from each geographic region was identified. In the second stage, one city was randomly selected within each chosen province. In the third stage, one university was randomly selected within each city. Finally, one academic department within each university was randomly chosen as the sampling unit.

All students enrolled in the selected departments were invited to participate in the study. This multistage stratified approach was designed to improve external validity by reducing regional clustering effects while maintaining feasibility.

Based on an anticipated target of approximately 100 participants per university, 110 questionnaires were distributed at each of the five selected universities (total distributed = 550). After excluding incomplete or invalid responses, 508 questionnaires were retained for final analysis, yielding a valid response rate of 92.4%.

Given that the present study employs a moderated mediation model including an interaction term, sample size adequacy was evaluated in relation to statistical power for detecting interaction effects. Prior methodological research suggests that interaction effects in regression analyses are often small and require relatively large samples (Aiken and West, 1991; Cohen, 1988).

To further assess adequacy, a *post hoc* power analysis was conducted using G*Power 3.1 (Faul et al., 2009). Assuming a small effect size (*f*^2^ = 0.02), an alpha level of 0.05, and the number of predictors included in the model (*k* = 8), the achieved statistical power exceeded 0.80 with the present sample size (*N* = 508), indicating that the study was sufficiently powered to detect small-to-moderate effects in the moderated mediation analysis.

The final sample consisted of students from five universities, each representing one academic department, thereby maintaining consistency with the predefined sampling framework.

Data collection was outsourced to a professional research agency, S Research Institute, which was commissioned to implement the sampling and data collection procedures strictly in accordance with the predefined methodological protocol. The agency confirmed adherence to the stratified sampling design and ensured that recruitment procedures were conducted in compliance with established guidelines.

To ensure data quality, the research agency implemented standardized data collection protocols and monitored response completeness. Invalid or incomplete responses were excluded prior to analysis, and compliance with the predefined sampling procedures was carefully monitored throughout the process.

Survey administration was conducted online. Participants received detailed information outlining the study objectives, procedures, confidentiality safeguards, and voluntary participation principles prior to accessing the questionnaire.

Prior to participation, all respondents provided electronic informed consent. Participants were informed that their participation was voluntary and that they could withdraw at any time without penalty. The study protocol was reviewed and approved by the Institutional Review Board of the affiliated university (Approval No. HS26-0213-06), in accordance with established ethical guidelines for research involving human subjects.

The final sample (*N* = 508) included 40.2% male and 59.8% female participants. Regarding age distribution, 23.2% were 18 years old, 47.8% were 19 years old, 22.6% were 20 years old, and 6.5% were 21 years or older, with 19-year-old students representing the largest age group. The relatively concentrated age range reflects the typical demographic composition of undergraduate populations in China.

### 3.2 Research tools

#### 3.2.1 Physical exercise

Physical exercise was assessed using the Physical Activity Rating Scale-3 (PARS-3), originally developed by Hashimoto (1975) and later adapted for Chinese populations by Liang (1994). The scale assesses three dimensions of physical activity: exercise intensity, duration, and frequency.

In the present study, the mean score of the three items, each rated on a 5-point Likert scale, was used for analysis. This scoring approach was adopted to ensure measurement consistency across study constructs and to enhance estimation stability in structural equation modeling, as multiplicative scoring methods may introduce non-normality and scaling distortions.

Importantly, the PARS-3 was conceptualized as a behavioral index rather than a reflective latent construct. The three components—intensity, duration, and frequency—represent distinct but complementary aspects of physical exercise behavior, and are not assumed to reflect a single underlying latent factor. Therefore, conventional criteria used for reflective measurement models, such as average variance extracted (AVE), may be less appropriate, and relatively lower AVE values are considered acceptable in this context.

Higher scores indicate greater levels of habitual physical exercise.

Internal consistency reliability was assessed using Cronbach’s *α*. The three items demonstrated acceptable internal consistency (*α* = 0.706), which is considered adequate given the scale’s three-item structure. Consistent with prior studies (Ding et al., 2021; Yue et al., 2022), the PARS-3 was treated as a behavioral index rather than a reflective latent construct.

#### 3.2.2 Grit

The Grit Scale developed by Duckworth and Quinn (2009) and translated by Xie et al. (2017) was used in this study. The scale consists of eight items, with four items measuring consistency of interest and four measuring perseverance of effort. Responses were rated on a 5-point Likert scale, with higher scores indicating greater levels of grit. The scale demonstrated acceptable internal consistency, with Cronbach’s alpha coefficients of 0.893 and 0.838 for its subdimensions.

#### 3.2.3 Peer relationship

Peer relationships were assessed using the Loneliness and Social Dissatisfaction Scale developed by Asher et al. (1984). The scale consists of 16 items rated on a 5-point Likert scale. Higher scores indicate lower levels of loneliness and more positive peer relationships. The scale demonstrated acceptable internal consistency, with Cronbach’s alpha coefficients of 0.933 and 0.872 for its subdimensions.

#### 3.2.4 Hope

The Hope Scale developed by Snyder et al. (1991) and translated by Shi and Tian (2009) was used in this study. The scale consists of eight items, with four items measuring pathways thinking and four measuring agency thinking. Responses were rated on a 5-point Likert scale, with higher scores indicating higher levels of hope. Although Snyder’s Hope Theory conceptualizes hope as two related dimensions—agency and pathways—the present findings supported a unidimensional structure in this sample. Nevertheless, future research should compare one-factor and two-factor models more explicitly to determine whether the dimensional structure of hope varies across cultural and developmental contexts. The scale demonstrated acceptable internal consistency, as indicated by Cronbach’s alpha coefficients of 0.942.

#### 3.2.5 Happiness

This study used the happiness scale developed by Campbell et al. (1976). The scale originally consists of eight items rated on a 7-point Likert scale, with higher scores indicating greater happiness. The scale demonstrated acceptable internal consistency, as indicated by Cronbach’s alpha coefficients of 0.947.

### 3.3 Data analysis

All statistical analyses were conducted using IBM SPSS Statistics version 25.0, AMOS version 23.0, and PROCESS macro version 4.2 (Hayes, 2022). Statistical significance was evaluated at the 0.05 level unless otherwise specified.

Descriptive statistics and frequency analyses were first performed to summarize participants’ demographic characteristics and to assess the distributional properties of the study variables. Skewness and kurtosis values were examined to verify the assumption of normality.

To evaluate construct validity, both exploratory factor analysis (EFA) and confirmatory factor analysis (CFA) were conducted. EFA was performed using principal axis factoring with oblique rotation to explore the underlying factor structure. Subsequently, CFA was conducted using maximum likelihood estimation to validate the measurement model. Model fit was evaluated using multiple goodness-of-fit indices, including *χ*^2^/df, CFI, TLI, and RMSEA, following commonly recommended cutoff criteria (Hu and Bentler, 1999; Marsh et al., 2004).

Internal consistency reliability was assessed using Cronbach’s alpha and McDonald’s omega coefficients. In addition, composite reliability (CR) and average variance extracted (AVE) were examined to establish convergent validity.

To assess potential common method bias (CMB), both Harman’s single-factor test and the Common Latent Factor (CLF) approach were employed. While Harman’s test provides a preliminary diagnostic, the CLF technique was used to more rigorously examine the potential influence of common method variance within the CFA framework.

Pearson’s correlation analyses were conducted to examine bivariate associations among the key variables.

Following CFA, composite scores were computed for each construct and used in subsequent analyses. This two-step approach—validating measurement properties through CFA and then testing structural relationships using observed composite variables—is commonly adopted in behavioral research and allows for efficient estimation of complex conditional process models.

To test the hypothesized moderated mediation model, Model 83 of the PROCESS macro was applied. All predictor variables were mean-centered prior to analysis to reduce potential multicollinearity. A bootstrapping procedure with 5,000 resamples was used to generate bias-corrected 95% confidence intervals (CIs) for indirect and conditional indirect effects. Conditional indirect effects were estimated at low (−1 SD), mean, and high (+1 SD) levels of the moderator. Effects were considered statistically significant when the 95% confidence interval did not include zero.

## 4 Research results

### 4.1 Measurement model

To assess the structural validity of the measurement scales, exploratory factor analyses (EFA) and confirmatory factor analyses (CFA) were conducted for all constructs. The results generally supported the proposed factor structures, with acceptable sampling adequacy (KMO values ranging from 0.764 to 0.930) and significant Bartlett’s tests (*p* < 0.001).

For grit and peer relationship, EFA results supported two-factor structures, and subsequent second-order CFA confirmed that the first-order factors loaded significantly onto higher-order constructs. In contrast, hope and happiness demonstrated unidimensional structures, consistent with theoretical expectations and prior empirical findings.

The CFA results indicated acceptable model fit across all constructs (see Table 1), with fit indices meeting recommended thresholds (CFI = 0.951–0.994, TLI = 0.941–0.983, RMSEA = 0.071–0.077; Hu and Bentler, 1999). Specifically, all constructs demonstrated satisfactory model fit, supporting the adequacy of the measurement model.

**Table 1:** Model fit indices.

Construct	CMIN	CMIN/DF	GFI	TLI	CFI	RMSEA
Grit	40.111^***^	3.646	0.978	0.974	0.986	0.072
Peer relationship	350.592^***^	3.541	0.924	0.941	0.951	0.071
Hope	760,411^***^	1.022	0.966	0.976	0.983	0.077
Happiness	26.91^***^	3.844	0.985	0.983	0.994	0.075

CMIN = chi-square statistic; CMIN/DF = chi-square divided by degrees of freedom; GFI = goodness-of-fit index; TLI = Tucker–Lewis index; CFI = comparative fit index; RMSEA = root mean square error of approximation. ^***^p < 0.001. Values of CFI and TLI above 0.90 and RMSEA below 0.08 indicate acceptable model fit.

As summarized in Table 2, convergent validity and reliability were generally supported. Composite reliability (CR) values ranged from 0.88 to 0.96, exceeding the recommended threshold of 0.70, and most average variance extracted (AVE) values were above the recommended level of 0.50 (Fornell and Larcker, 1981), with the exception of physical exercise, which was slightly below the criterion. Internal consistency was further confirmed by Cronbach’s *α* and McDonald’s *ω* coefficients, all of which exceeded 0.70.

**Table 2:** Measurement model results.

Construct	Factor structure	KMO	AVE	CR	Cronbach’s *α*	McDonald’s *ω*
Physical exercise	–	0.764	0.469	0.642	0.706	0.642
Grit	Consistency of interest	0.778	0.607	0.884	0.893	0.884
Grit	Perseverance of effort	0.778	0.842	0.914	0.838	0.914
Peer relationship	Factor1	0.928	0.580	0.993	0.933	0.993
Peer relationship	Factor2	0.928	0.537	0.914	0.872	0.914
Hope	–	0.930	0.674	0.958	0.942	0.958
Happiness	–	0.904	0.721	0.958	0.947	0.958

AVE = average variance extracted; CR = composite reliability. For multidimensional constructs (grit and peer relationship), values are reported separately for each factor.

Based on these results, all constructs demonstrated acceptable reliability and validity. Accordingly, composite scores were computed and used in subsequent analyses.

### 4.2 Common method bias test

To mitigate potential common method bias (CMB), both procedural and statistical remedies were applied, consistent with recommendations in prior methodological literature (Podsakoff et al., 2003, 2012). Procedurally, respondent anonymity was assured, and measurement items were presented in a randomized order across constructs to reduce evaluation apprehension and consistency motifs.

As an initial diagnostic, Harman’s single-factor test was conducted by entering all measurement items into an unrotated exploratory factor analysis. The first factor accounted for 32.09% of the total variance, which is below the 50% threshold suggested by Podsakoff et al. (2003). Although Harman’s test has been criticized for its limited sensitivity in detecting method bias (Podsakoff et al., 2012; Howard and Boudreaux, 2024), the results provide preliminary evidence that a single latent factor does not dominate the covariance structure.

To further examine the potential impact of common method variance (CMV), a Common Latent Factor (CLF) was incorporated into the measurement model. The inclusion of the CLF resulted in modest improvements in model fit (CMIN/DF = 3.785, CFI = 0.919, TLI = 0.903, RMSEA = 0.074) relative to the baseline model (CMIN/DF = 4.705, CFI = 0.885, TLI = 0.875, RMSEA = 0.085). While this improvement suggests the possibility of some shared method variance, the magnitude of change was minimal.

Importantly, the standardized factor loadings and substantive structural path coefficients remained largely stable after controlling for the CLF, with no meaningful changes in magnitude or statistical significance. This stability indicates that common method variance does not materially bias the estimated relationships among the focal constructs. This pattern is consistent with prior research suggesting that minor improvements in model fit after including a CLF do not necessarily indicate substantial common method bias.

In addition, to further assess the potential impact of common method bias, variance inflation factor (VIF) values were examined following the full collinearity approach. The VIF values ranged from 1.095 to 1.462, all of which were well below the recommended threshold of 3.3. These results indicate that common method bias is unlikely to pose a serious threat to the validity of the findings.

Taken together, these results provide converging evidence that common method bias is unlikely to substantially influence the observed relationships or threaten the validity of the study’s conclusions.

### 4.3 Correlation between main variables

Correlation analyses indicated that most study variables were positively and significantly associated, as shown in Table 3. The highest correlation was observed between hope and happiness (*r* = 0.721, *p* < 0.01). However, as none of the intercorrelations exceeded the conservative threshold of 0.80 (Hair et al., 2010; Tabachnick and Fidell, 2019), multicollinearity was not considered a serious concern. Skewness and kurtosis values fell within acceptable ranges (|skewness| < 2; |kurtosis| < 7), supporting the assumption of normality (West et al., 1995).

**Table 3:** Correlation and descriptive statistics.

(*N* = 508)					
Categories	1	2	3	4	5
1. Physical exercise	1				
2. Grit	0.088^*^	1			
3. Peer relationship	0.218^**^	0.370^**^	1		
4. Hope	0.266^**^	−0.009	0.413^**^		
5. Happiness	0.168^**^	−0.059	0.373^**^	0.721^**^	
M	3.076	2.975	3.595	3.671	3.898
SD	0.961	0.829	0.600	0.7048	0.774
Skewness	0.028	−0.304	0.214	0.024	−0.652
Kurtosis	−0.833	0.538	0.091	0.543	1.315

^*^p < 0.05, ^**^p < 0.01.

These findings provide preliminary evidence in support of the proposed relationships among physical exercise, peer relationships, hope, grit, and happiness, thereby justifying subsequent mediation and moderated mediation analyses.

### 4.4 Moderated mediation effect of peer relationship

**Figure 2:**
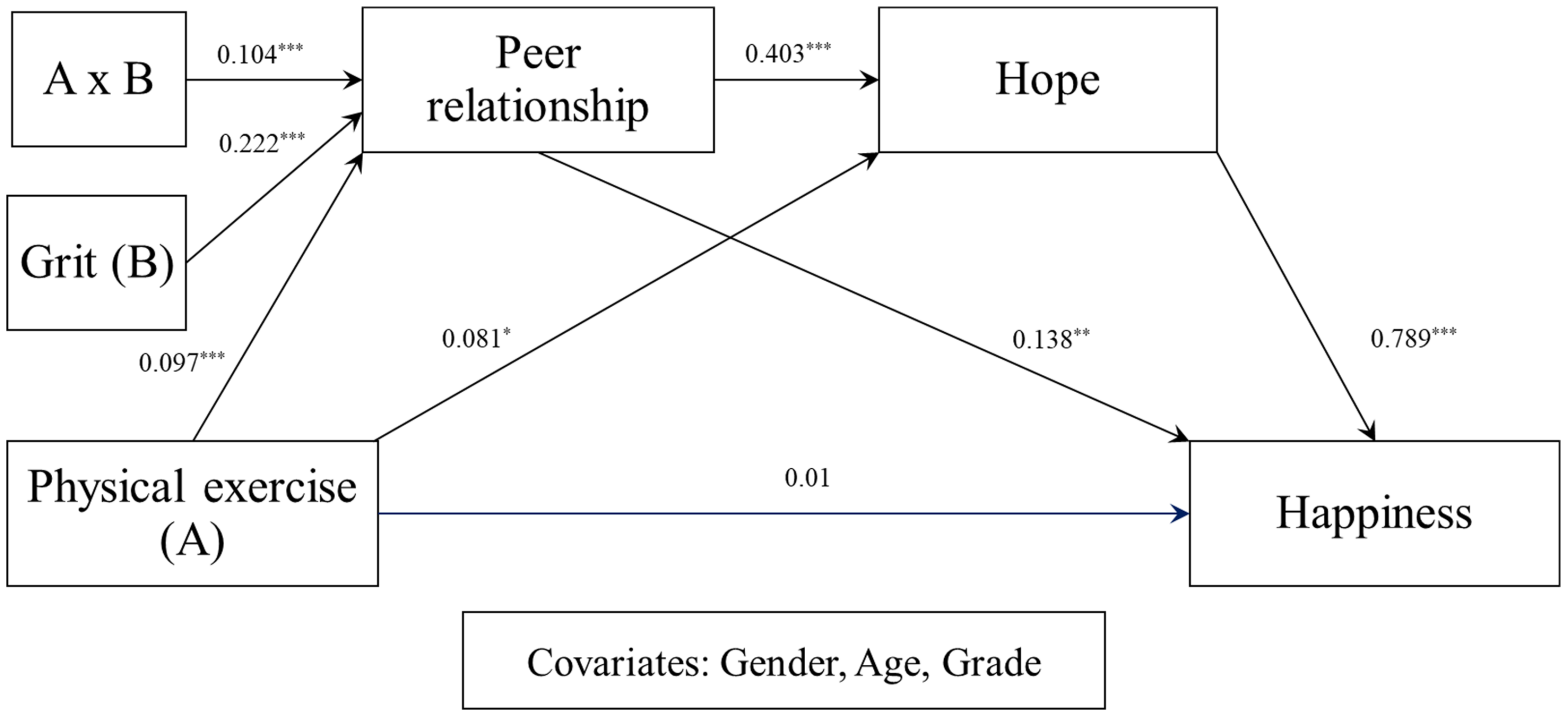
Statistical model of moderated mediation effect of grit.

**Figure 3:**
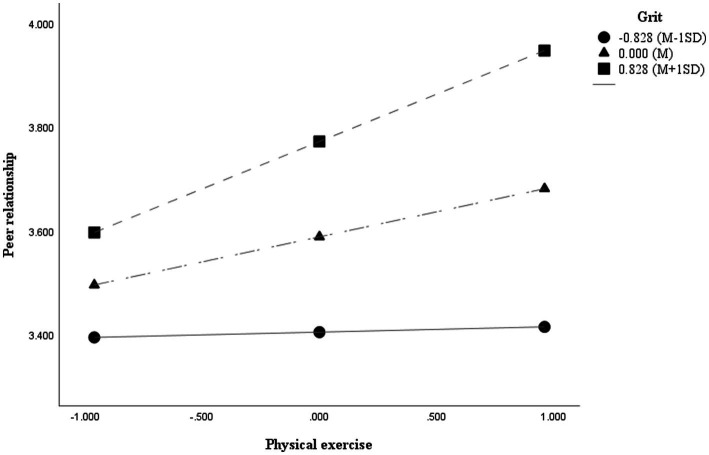
Moderating effect of grit on the relationship between physical exercise and peer relationship.

In the present study, a moderated serial mediation model was tested to examine whether grit conditions the indirect relationship between physical exercise and happiness through peer relationships and hope, using PROCESS Macro Model 83 (Hayes, 2022). Physical exercise and grit were mean-centered prior to analysis, and gender, age, and grade were included as control variables. The results are presented in Figures 2, 3 and Tables 4, 5.

**Table 4:** Analysis of moderating effect of grit.

Classification		DV: Peer relationship			DV: Hope			DV: Happiness		
		*B*	SE	*t* value	*B*	SE	*t* value	*B*	SE	*t* value
Constant		3.107	0.324	9.588^***^	1.198	0.402	2.978^**^	0.494	0.346	1.428
IV	Physical exercise	0.097	0.028	3.437^***^	0.081	0.032	2.519^*^	0.010	0.028	0.376
M1	Peer relationship				0.403	0.047	8.504^***^	0.138	0.043	3.183^**^
M2	Hope				.			0.789	0.038	20.714^***^
Moderator	Grit	0.222	0.031	7.119^***^						
Interaction	Physical exercise × Grit	0.104	0.029	3.583^***^						
Higher order test	*R*^2^ change	0.020								
	*F*	12.838^***^								
Covariates	Gender	0.136	0.055	2.475*	0.262	0.063	4.140^***^	−0.206	0.055	−3.750^***^
	Age	−0.028	0.026	−1.057	−0.033	0.030	−1.078	0.041	0.026	1.583
	Grade	0.483	0.316	1.530	0.985	0.357	2.757^**^	0.010	0.307	0.031
Model summary	*R* ^2^	0.214			0.242			0.544		
	*F*	22.792^***^			32.129^***^			99.608^***^		
Mediating variable model (DV: Peer relationship)										
Conditional effects of the physical exercise at values of the grit										
Grit		*B*		se	*t* value		LLCI		ULCI	
−0.828 (M − 1SD)		0.011		0.034	0.313		−0.056		0.077	
0.000 (M)		0.097		0.028	3.437^***^		0.041		0.152	
0.828 (M + 1SD)		0.183		0.040	4.580^***^		0.104		0.261	
Moderator value(s) defining Johnson-Neyman significance region(s)										
Value				% below			% above			
−0.391				23.819			76.181			
Conditional effect of focal predictor at values of the moderator										
Grit		B	se		*t* value		LLCI		ULCI	
−1.975		−0.109	0.060		−1.825		−0.225		0.008	
:										
−0.575		0.037	0.030		1.221		−0.023		0.096	
−0.391		0.056	0.029		1.965		0.000		0.112	
−0.375		0.058	0.028		2.030^*^		0.002		0.114	
:										
2.025		0.307	0.069		4.438^***^		0.171		0.443	

M1 = Mediator 1, M2 = Mediator 2, IV=Independent Variable, DV: Dependent Variable; ^*^p < 0.05, ^**^p < 0.01, ^***^p < 0.001, LLCI = Bootstrap lower bound within 95% confidence interval, ULCI = Bootstrap upper bound within 95% confidence interval.

**Table 5:** Analysis of direct effect and conditional indirect effect.

Direct effect (Physical exercise → Happiness)				
*B*	se	*t* value	LLCI	ULCI
0.010	0.028	0.376	−0.044	0.065
Conditional indirect effects of X on Y				
Physical exercise → Peer relationship→ Hope → Happiness				
Grit	B	BootSE	BootLLCI	BootULCI
−0.828 (M − 1SD)	0.003	0.014	−0.025	0.029
0.000 (M)	0.031	0.010	0.012	0.052
0.828 (M + 1SD)	0.058	0.016	0.029	0.092
Index of moderated mediation				
Grit	Index	BootSE	BootLLCI	BootULCI
	0.033	0.013	0.010	0.061

LLCI = Lower limit within the 95% confidence interval of the bootstrapped indirect effect, ULCI = Upper limit within the 95% confidence interval of the bootstrapped indirect effect.

First, in the model predicting peer relationships, physical exercise was significantly related to peer relationships (*B* = 0.097, *p* < 0.001), indicating that higher levels of physical exercise are associated with more positive peer relationships. Grit also showed a positive association (*B* = 0.222, *p* < 0.001). Importantly, the interaction between physical exercise and grit was statistically significant (*B* = 0.104, *p* < 0.001), suggesting that the association between physical exercise and peer relationships varies depending on the level of grit. The increase in explained variance attributable to the interaction was significant (Δ*R*^2^ = 0.020, *p* < 0.001), confirming the moderating role of grit in the first-stage pathway.

Conditional effects analysis further showed that the positive association between physical exercise and peer relationships was not significant at low levels of grit (−1 SD), but became significant at mean and high (+1 SD) levels of grit. The Johnson–Neyman analysis indicated that this effect was significant when grit exceeded −0.391, corresponding to approximately 76.2% of the sample. These findings suggest that individuals with higher levels of grit are more likely to translate physical exercise into enhanced peer relationships.

In the subsequent model predicting hope, peer relationships were positively associated with hope (*B* = 0.403, *p* < 0.001), indicating that more positive peer relationships are associated with higher levels of hope. Physical exercise also showed a smaller but significant positive association (*B* = 0.081, *p* < 0.05). In the final model predicting happiness, hope showed a positive association with happiness (*B* = 0.789, *p* < 0.001), and peer relationships also significantly predicted happiness (*B* = 0.138, *p* < 0.01). However, the direct effect of physical exercise on happiness was not significant (*B* = 0.010, *p* > 0.05), suggesting that its influence operates primarily through indirect pathways.

Taken together, these results support the proposed sequential mediation mechanism, whereby physical exercise is associated with increased peer relationships, which in turn enhance hope, ultimately contributing to greater happiness. Furthermore, the findings indicate that this indirect pathway is contingent upon levels of grit, with stronger effects observed at higher levels of grit. The final model accounted for 54.4% of the variance in happiness (*R*^2^ = 0.544, *p* < 0.001), indicating substantial explanatory power.

Figure 3 shows that as physical exercise increases, peer relationship tends to improve overall. In particular, the slope becomes steeper at higher levels of grit, indicating that the association between physical exercise and peer relationships is strengthened. In contrast, among individuals with low levels of grit, the impact of physical exercise appears to be minimal.

The direct and moderated mediation effects are presented in Table 5. The analysis of the direct effect indicated that physical exercise did not have a statistically significant direct effect on happiness (*B* = 0.010, 95% CI [−0.044, 0.065]). This finding suggests that physical exercise is not directly related to happiness, but rather operates indirectly through the proposed mediating variables.

The analysis of conditional indirect effects revealed that the magnitude of the indirect effect varied as a function of grit. At low levels of grit (−1 SD), the conditional indirect effect was not statistically significant, as the confidence interval included zero. However, at the mean and high (+1 SD) levels of grit, the conditional indirect effects were statistically significant. Notably, as grit increased, the strength of the indirect pathway from physical exercise to happiness through peer relationships and hope became progressively stronger.

The index of moderated mediation was 0.033, and the 95% bootstrap confidence interval [0.009, 0.061] did not include zero, indicating a statistically significant moderated mediation effect. These findings demonstrate that the indirect effect of physical exercise on happiness through peer relationships and hope is contingent upon levels of grit, with stronger indirect effects observed at higher levels of grit. This pattern is consistent with the proposed moderated serial mediation model, in which grit strengthens the indirect pathway linking physical exercise to happiness.

## 5 Discussion and conclusion

The present study extends the literature on youth development and wellbeing by demonstrating that physical exercise, grit, peer relationship, hope, and happiness are positively and significantly interrelated. Among these associations, the strongest correlation was observed between hope and happiness, underscoring the central role of hope as a proximal cognitive-motivational determinant of subjective wellbeing. Consistent with Hope Theory, hope functions as a goal-directed cognitive system that enables individuals to sustain motivation and generate effective pathways toward desired outcomes, thereby enhancing life satisfaction and positive affect.

The positive associations involving physical exercise can be understood through the broaden-and-build theory of positive emotions. Engagement in physical activity may foster positive affect and adaptive coping, which in turn facilitate the accumulation of enduring personal and social resources. In university contexts, where exercise is often socially embedded, this process may be particularly effective in fostering both relational resources (peer relationship) and cognitive-motivational resources (hope). In addition, grit appears to play a facilitative role by enhancing individuals’ persistence in both physical and social domains, thereby increasing the likelihood of accumulating beneficial psychological resources.

Importantly, the findings support a sequential mechanism in which physical exercise is associated with happiness through peer relationships and hope. This pattern suggests an indirect-only mediation, in which the indirect effects are statistically significant while the direct effect is not. This indicates that physical exercise is linked to happiness primarily through relational and cognitive-motivational pathways rather than through a direct association. This interpretation should be made with caution, however, as the cross-sectional design does not permit strong causal inferences.

The moderated serial mediation analysis further revealed that the strength of the indirect pathway varies as a function of grit. Specifically, the indirect effects were significant at moderate and high levels of grit but not at low levels. This finding suggests that grit operates as a boundary condition that influences the extent to which individuals can translate behavioral engagement into relational and cognitive resources. Individuals higher in grit may be more likely to sustain participation in physical and social activities, thereby strengthening the sequential pathway from exercise to happiness.

At the same time, alternative explanations and potential reciprocal relationships should be considered. Although the proposed model assumes that physical exercise contributes to happiness through peer relationships and hope, it is also plausible that individuals with higher levels of happiness are more likely to engage in physical activity and develop stronger social relationships. Similarly, hope may function not only as an outcome of social experiences but also as a precursor that promotes greater engagement in both physical and interpersonal domains. These possibilities highlight the potential bidirectional nature of the relationships among the study variables and underscore the need for longitudinal or experimental research to establish causal ordering.

The findings should also be interpreted within their cultural context. In collectivistic societies such as China, interpersonal relationships play a central role in shaping psychological wellbeing, with strong emphasis placed on social harmony and connectedness. As a result, the associations among physical exercise, peer relationships, hope, and happiness may be more tightly interconnected, with relational processes serving as key pathways through which behavioral engagement influences wellbeing. In contrast, in more individualistic contexts, psychological wellbeing may be more strongly influenced by personal autonomy and individual achievement, and physical exercise may exert more direct effects on happiness. Therefore, caution is warranted in generalizing the present findings beyond collectivistic populations.

From a practical perspective, the results suggest that universities should move beyond simply promoting physical exercise and instead design socially embedded exercise programs that facilitate repeated peer interaction. Group-based activities, peer-led programs, and collaborative exercise formats may be particularly effective in strengthening peer relationships. Additionally, integrating goal-setting strategies and feedback mechanisms may enhance hope, thereby helping students translate physical activity into sustained psychological benefits. Given that the strength of these relationships varies across levels of grit, interventions should also consider individual differences and provide additional support for students with lower levels of perseverance.

Despite its contributions, this study has several limitations. First, the cross-sectional design limits causal inference. Second, reliance on self-report measures may introduce bias. Third, the sample is restricted to Chinese university students, which may limit generalizability. Fourth, alternative structural models were not tested, and future studies should examine competing models using more advanced analytical approaches. Finally, further validation using longitudinal designs and diverse samples is needed to confirm the robustness of the proposed relationships.

In conclusion, the present study provides an integrated framework linking physical exercise, peer relationships, hope, and happiness, while highlighting the conditional role of grit. By demonstrating that behavioral engagement influences wellbeing primarily through relational and cognitive-motivational pathways, the findings contribute to a more nuanced understanding of psychological wellbeing in emerging adulthood.

## Data Availability

The original contributions presented in the study are included in the article/Supplementary material, further inquiries can be directed to the corresponding author.
