# Supplementary material for: Effects of physical exercise on the happiness of Chinese university students: the moderated mediation model of grit through peer relationship and hope
Source: Front Psychol. 2026 May 22;17:1822698. doi: 10.3389/fpsyg.2026.1822698 (PMC13236558; doi:10.3389/fpsyg.2026.1822698)
Supplement: Supplementary file 1 [file Data_Sheet_1.pdf]

Reduced Scale

Run MATRIX procedure:

*************** PROCESS Procedure for SPSS Version 4.2 beta ***************

Written by Andrew F. Hayes, Ph.D.   www.afhayes.com
Documentation available in Hayes (2022). www.guilford.com/p/hayes3

****
Model   : 83
Y   : M_happin
X   : M_Volume
M1   : M_Peer
M2   : M_hope
W   : M_Grit

Covariates:
grade   gender   age

Sample
Size: 508

****
OUTCOME VARIABLE:
M_Peer

Model Summary

R	R-sq	MSE	F	df1	df2	p
.4631	.2144	.2863	22.7924	6.0000	501.0000	.0000

Model

coeff   se   t   p   LLCI
ULCI

constant	3.1069	.3240	9.5879	.0000	2.4702	3.7435
M_Volume	.0966	.0281	3.4374	.0006	.0414	.1519
M_Grit	.2220	.0312	7.1187	.0000	.1608	.2833
Int_1	.1039	.0290	3.5830	.0004	.0469	.1608
grade	.4833	.3158	1.5301	.1266	-.1373	1.1038
gender	.1357	.0548	2.4746	.0137	.0280	.2435
age	-.0278	.0263	-1.0567	.2912	-.0796	.0239

Product terms key:

Int_1 : M_Volume x M_Grit

Test(s) of highest order unconditional interaction(s):

	R2-chng	F	df1	df2	p
X*W	.0201	12.8381	1.0000	501.0000	.0004

Focal predict: M_Volume (X)

Mod var: M_Grit (W)

Conditional effects of the focal predictor at values of the moderator(s):

M_Grit	Effect	se	t	p	LLCI	ULCI
-.8284	.0106	.0338	.3132	.7542	-.0558	.0770
.0000	.0966	.0281	3.4374	.0006	.0414	.1519
.8284	.1827	.0399	4.5798	.0000	.1043	.2610

Moderator value(s) defining Johnson-Neyman significance region(s):

Value	% below	% above
-.3910	23.8189	76.1811

Conditional effect of focal predictor at values of the moderator:

M_Grit	Effect	se	t	p	LLCI	ULCI
-1.9752	-.1085	.0595	-1.8253	.0686	-.2253	.0083
-1.7752	-.0877	.0544	-1.6132	.1073	-.1946	.0191
-1.5752	-.0670	.0495	-1.3532	.1766	-.1642	.0303
-1.3752	-.0462	.0448	-1.0310	.3030	-.1342	.0418
-1.1752	-.0254	.0404	-.6292	.5295	-.1048	.0540

-.9752	-.0047	.0364	-.1278	.8984	-.0762	.0669
-.7752	.0161	.0329	.4892	.6249	-.0486	.0809
-.5752	.0369	.0302	1.2211	.2226	-.0225	.0963
-.3910	.0560	.0285	1.9647	.0500	.0000	.1120
-.3752	.0577	.0284	2.0297	.0429	.0018	.1135
-.1752	.0784	.0277	2.8290	.0049	.0240	.1329
.0248	.0992	.0282	3.5130	.0005	.0437	.1547
.2248	.1200	.0299	4.0138	.0001	.0613	.1787
.4248	.1408	.0325	4.3297	.0000	.0769	.2046
.6248	.1615	.0359	4.5020	.0000	.0910	.2320
.8248	.1823	.0398	4.5790	.0000	.1041	.2605
1.0248	.2031	.0442	4.5988	.0000	.1163	.2898
1.2248	.2238	.0488	4.5864	.0000	.1280	.3197
1.4248	.2446	.0537	4.5571	.0000	.1392	.3501
1.6248	.2654	.0587	4.5196	.0000	.1500	.3808
1.8248	.2862	.0639	4.4791	.0000	.1606	.4117
2.0248	.3069	.0692	4.4382	.0000	.1711	.4428

Data for visualizing the conditional effect of the focal predictor:
Paste text below into a SPSS syntax window and execute to produce plot.

```
DATA LIST FREE/
   M_Volume    M_Grit      M_Peer      .
BEGIN DATA.
     -.9605     -.8284     3.3936
      .0000     -.8284     3.4037
      .9605     -.8284     3.4139
     -.9605      .0000     3.4949
      .0000      .0000     3.5877
      .9605      .0000     3.6805
     -.9605      .8284     3.5962
      .0000      .8284     3.7716
      .9605      .8284     3.9471
END DATA.
GRAPH/SCATTERPLOT=
 M_Volume WITH      M_Peer    BY         M_Grit     .
```

****
OUTCOME VARIABLE:
M_hope

Model Summary

R	R-sq	MSE	F	df1	df2	p
.4924	.2424	.3801	32.1293	5.0000	502.0000	.0000

Model

	coeff	se	t	p	LLCI	ULCI
constant	1.1976	.4022	2.9778	.0030	.4075	1.9878
M_Volume	.0811	.0322	2.5191	.0121	.0178	.1444
M_Peer	.4026	.0473	8.5035	.0000	.3096	.4956
grade	.9845	.3571	2.7566	.0061	.2828	1.6862
gender	.2619	.0633	4.1395	.0000	.1376	.3861
age	-.0325	.0302	-1.0778	.2817	-.0919	.0268

****
OUTCOME VARIABLE:
M_happin

Model Summary

R	R-sq	MSE	F	df1	df2	p
.7376	.5440	.2765	99.6077	6.0000	501.0000	.0000

Model

	coeff	se	t	p	LLCI	ULCI
constant	.4941	.3461	1.4278	.1540	-.1858	1.1740
M_Volume	.0104	.0276	.3758	.7073	-.0439	.0647
M_Peer	.1375	.0432	3.1834	.0015	.0526	.2224
M_hope	.7886	.0381	20.7141	.0000	.7138	.8634
grade	.0096	.3069	.0312	.9751	-.5934	.6126
gender	-.2058	.0549	-3.7504	.0002	-.3136	-.0980

age	.0408	.0258	1.5825	.1142	-.0099	.0915

****************** DIRECT AND INDIRECT EFFECTS OF X ON Y *****************

Direct effect of X on Y

Effect	se	t	p	LLCI	ULCI
.0104	.0276	.3758	.7073	-.0439	.0647

Conditional and unconditional indirect effects of X on Y:

INDIRECT EFFECT:

M_Volume -> M_Peer -> M_happin

M_Grit	Effect	BootSE	BootLLCI	BootULCI
-.8284	.0015	.0063	-.0121	.0139
.0000	.0133	.0058	.0028	.0254
.8284	.0251	.0102	.0060	.0459

Index of moderated mediation:

	Index	BootSE	BootLLCI	BootULCI
M_Grit	.0143	.0075	.0019	.0311

Pairwise contrasts between conditional indirect effects (Effect1 minus Effect2)

Effect1	Effect2	Contrast	BootSE	BootLLCI	BootULCI
.0133	.0015	.0118	.0062	.0016	.0257
.0251	.0015	.0237	.0124	.0032	.0514
.0251	.0133	.0118	.0062	.0016	.0257

INDIRECT EFFECT:

M_Volume -> M_hope -> M_happin

Effect	BootSE	BootLLCI	BootULCI
.0640	.0286	.0088	.1208

INDIRECT EFFECT:

M_Volume -> M_Peer -> M_hope -> M_happin

M_Grit	Effect	BootSE	BootLLCI	BootULCI
-.8284	.0034	.0136	-.0247	.0292
.0000	.0307	.0102	.0117	.0516
.8284	.0580	.0160	.0286	.0917

Index of moderated mediation:

	Index	BootSE	BootLLCI	BootULCI
M_Grit	.0330	.0131	.0095	.0608

Pairwise contrasts between conditional indirect effects (Effect1 minus Effect2)

Effect1	Effect2	Contrast	BootSE	BootLLCI	BootULCI
.0307	.0034	.0273	.0108	.0079	.0504
.0580	.0034	.0546	.0216	.0158	.1007
.0580	.0307	.0273	.0108	.0079	.0504

********************** ANALYSIS NOTES AND ERRORS ***********************

Level of confidence for all confidence intervals in output:
95

Number of bootstrap samples for percentile bootstrap confidence intervals:
5000

W values in conditional tables are the mean and +/- SD from the mean.

NOTE: The following variables were mean centered prior to analysis:
M_Grit M_Volume

NOTE: Due to estimation problems, some bootstrap samples had to be replaced.
The number of times this happened was:
278

WARNING: Variables names longer than eight characters can produce incorrect output when some variables in the data file have the same first eight characters. Shorter variable names are recommended. By using this output, you are accepting all risk and consequences of interpreting or reporting results that may be incorrect.

------ END MATRIX -----

Full Scale

Run MATRIX procedure:

*************** PROCESS Procedure for SPSS Version 4.2 beta ***************

Written by Andrew F. Hayes, Ph.D. www.afhayes.com
Documentation available in Hayes (2022). www.guilford.com/p/hayes3

****
Model : 83
Y : T_happin
X : M_Volume
M1 : M_Peer
M2 : M_hope
W : T_Grit

Covariates:
gender age grade

Sample
Size: 508

****
OUTCOME VARIABLE:
M_Peer

Model Summary

R	R-sq	MSE	F	df1	df2	p
.5244	.2750	.2642	31.6736	6.0000	501.0000	.0000

Model

	coeff	se	t	p	LLCI	ULCI
constant	3.0440	.3095	9.8352	.0000	2.4359	3.6520
M_Volume	.0846	.0271	3.1257	.0019	.0314	.1378
T_Grit	.4145	.0418	9.9184	.0000	.3324	.4966
Int_1	.1294	.0391	3.3056	.0010	.0525	.2063
gender	.1103	.0529	2.0851	.0376	.0064	.2142
age	-.0252	.0253	-.9967	.3194	-.0750	.0245
grade	.5482	.3014	1.8190	.0695	-.0439	1.1402

Product terms key:

Int_1 : M_Volume x T_Grit

Test(s) of highest order unconditional interaction(s):

	R2-chng	F	df1	df2	p
X*W	.0158	10.9268	1.0000	501.0000	.0010

Focal predict: M_Volume (X)

Mod var: T_Grit (W)

Conditional effects of the focal predictor at values of the moderator(s):

T_Grit	Effect	se	t	p	LLCI	ULCI
-.5866	.0087	.0323	.2698	.7874	-.0548	.0723
.0000	.0846	.0271	3.1257	.0019	.0314	.1378
.5866	.1605	.0384	4.1813	.0000	.0851	.2359

Moderator value(s) defining Johnson-Neyman significance region(s):

Value	% below	% above
-.2425	31.1024	68.8976

Conditional effect of focal predictor at values of the moderator:

T_Grit	Effect	se	t	p	LLCI	ULCI
-1.3952	-.0959	.0566	-1.6933	.0910	-.2072	.0154
-1.2327	-.0749	.0511	-1.4649	.1436	-.1753	.0255
-1.0702	-.0538	.0458	-1.1755	.2403	-.1438	.0361

-.9077	-.0328	.0408	-.8043	.4216	-.1130	.0473
-.7452	-.0118	.0362	-.3255	.7450	-.0830	.0594
-.5827	.0092	.0323	.2862	.7748	-.0542	.0726
-.4202	.0303	.0292	1.0376	.2999	-.0270	.0876
-.2577	.0513	.0272	1.8842	.0601	-.0022	.1048
-.2425	.0533	.0271	1.9647	.0500	.0000	.1065
-.0952	.0723	.0267	2.7096	.0070	.0199	.1247
.0673	.0933	.0276	3.3758	.0008	.0390	.1477
.2298	.1144	.0300	3.8171	.0002	.0555	.1732
.3923	.1354	.0333	4.0605	.0001	.0699	.2009
.5548	.1564	.0375	4.1703	.0000	.0827	.2301
.7173	.1774	.0422	4.2026	.0000	.0945	.2604
.8798	.1985	.0473	4.1938	.0000	.1055	.2914
1.0423	.2195	.0527	4.1649	.0000	.1159	.3230
1.2048	.2405	.0583	4.1273	.0000	.1260	.3550
1.3673	.2615	.0640	4.0868	.0001	.1358	.3873
1.5298	.2826	.0698	4.0466	.0001	.1454	.4198
1.6923	.3036	.0757	4.0080	.0001	.1548	.4524
1.8548	.3246	.0817	3.9719	.0001	.1640	.4852

Data for visualizing the conditional effect of the focal predictor:
Paste text below into a SPSS syntax window and execute to produce plot.

```
DATA LIST FREE/
   M_Volume    T_Grit      M_Peer      .
BEGIN DATA.
     -.9605      -.5866      3.3339
      .0000      -.5866      3.3423
      .9605      -.5866      3.3507
     -.9605       .0000      3.5041
      .0000       .0000      3.5854
      .9605       .0000      3.6667
     -.9605       .5866      3.6743
      .0000       .5866      3.8285
      .9605       .5866      3.9827
END DATA.
```

GRAPH/SCATTERPLOT=
M_Volume WITH M_Peer BY T_Grit .

****
OUTCOME VARIABLE:
M_hope

Model Summary

R	R-sq	MSE	F	df1	df2	p
.4924	.2424	.3801	32.1293	5.0000	502.0000	.0000

Model

	coeff	se	t	p	LLCI	ULCI
constant	1.1976	.4022	2.9778	.0030	.4075	1.9878
M_Volume	.0811	.0322	2.5191	.0121	.0178	.1444
M_Peer	.4026	.0473	8.5035	.0000	.3096	.4956
gender	.2619	.0633	4.1395	.0000	.1376	.3861
age	-.0325	.0302	-1.0778	.2817	-.0919	.0268
grade	.9845	.3571	2.7566	.0061	.2828	1.6862

****
OUTCOME VARIABLE:
T_happin

Model Summary

R	R-sq	MSE	F	df1	df2	p
.7214	.5204	.2396	90.6029	6.0000	501.0000	.0000

Model

	coeff	se	t	p	LLCI	ULCI
constant	.8506	.3221	2.6408	.0085	.2178	1.4834
M_Volume	.0120	.0257	.4678	.6401	-.0385	.0626
M_Peer	.1928	.0402	4.7959	.0000	.1138	.2718
M_hope	.6674	.0354	18.8344	.0000	.5977	.7370
gender	-.1805	.0511	-3.5335	.0004	-.2808	-.0801
age	.0352	.0240	1.4670	.1430	-.0119	.0823

grade	-.1773	.2857	-.6207	.5351	-.7386	.3839

****************** DIRECT AND INDIRECT EFFECTS OF X ON Y *****************

Direct effect of X on Y

Effect	se	t	p	LLCI	ULCI
.0120	.0257	.4678	.6401	-.0385	.0626

Conditional and unconditional indirect effects of X on Y:

INDIRECT EFFECT:

M_Volume -> M_Peer -> T_happin

T_Grit	Effect	BootSE	BootLLCI	BootULCI
-.5866	.0017	.0078	-.0151	.0166
.0000	.0163	.0060	.0056	.0286
.5866	.0310	.0093	.0139	.0503

Index of moderated mediation:

	Index	BootSE	BootLLCI	BootULCI
T_Grit	.0249	.0105	.0071	.0482

INDIRECT EFFECT:

M_Volume -> M_hope -> T_happin

Effect	BootSE	BootLLCI	BootULCI
.0541	.0246	.0068	.1025

INDIRECT EFFECT:

M_Volume -> M_Peer -> M_hope -> T_happin

T_Grit	Effect	BootSE	BootLLCI	BootULCI
-.5866	.0023	.0106	-.0193	.0224
.0000	.0227	.0082	.0077	.0401
.5866	.0431	.0122	.0215	.0693

Index of moderated mediation:

	Index	BootSE	BootLLCI	BootULCI
T_Grit	.0348	.0135	.0108	.0640

********************** ANALYSIS NOTES AND ERRORS ************************

Level of confidence for all confidence intervals in output:
95

Number of bootstrap samples for percentile bootstrap confidence intervals:
5000

W values in conditional tables are the mean and +/- SD from the mean.

NOTE: The following variables were mean centered prior to analysis:
T_Grit M_Volume

NOTE: Due to estimation problems, some bootstrap samples had to be replaced.
The number of times this happened was:
255

WARNING: Variables names longer than eight characters can produce incorrect output when some variables in the data file have the same first eight characters. Shorter variable names are recommended. By using this output, you are accepting all risk and consequences of interpreting or reporting results that may be incorrect.

------ END MATRIX -----
